# The CCL20-CCR6 Axis in Cancer Progression

**DOI:** 10.3390/ijms21155186

**Published:** 2020-07-22

**Authors:** Suguru Kadomoto, Kouji Izumi, Atsushi Mizokami

**Affiliations:** Department of Integrative Cancer Therapy and Urology, Kanazawa University Graduate School of Medical Science, 13-1 Takara-machi, Kanazawa 920-8641, Ishikawa, Japan; 32f3k8@bma.biglobe.ne.jp (S.K.); mizokami@staff.kanazawa-u.ac.jp (A.M.)

**Keywords:** chemokines, CCL20-CCR6 axis, cancer progression

## Abstract

Chemokines, which are basic proteins that exert their effects via G protein-coupled receptors and a subset of the cytokine family, are mediators deeply involved in leukocyte migration during an inflammatory reaction. Chemokine (C-C motif) ligand 20 (CCL20), also known as macrophage inflammatory protein (MIP)-3α, liver activation regulated chemokine (LARC), and Exodus-1, is a small protein that is physiologically expressed in the liver, colon, and skin, is involved in tissue inflammation and homeostasis, and has a specific receptor **C-C** chemokine receptor 6 (CCR6). The CCL20-CCR6 axis has long been known to be involved in inflammatory and infectious diseases, such as rheumatoid arthritis and human immunodeficiency virus infections. Recently, however, reports have shown that the CCL20-CCR6 axis is associated with several cancers, including hepatocellular carcinoma, colorectal cancer, breast cancer, pancreatic cancer, cervical cancer, and kidney cancer. The CCL20-CCR6 axis promotes cancer progression directly by enhancing migration and proliferation of cancer cells and indirectly by remodeling the tumor microenvironment through immune cell control. The present article reviewed the role of the CCL20-CCR6 axis in cancer progression and its potential as a therapeutic target.

## 1. Introduction

Cytokines are proteins produced by various cells, including immunocompetent cells that exert limited physiological activity. Most cytokines are soluble extracellular proteins with small molecular weights (5–20 kDa) that act by binding to specific receptors on target cells. Cytokines not only play important roles in the immune system, such as regulating the balance between humoral and cellular immunity, but also act on a wide range of cells, such as endothelial cells and fibroblasts. Cytokines sometimes damage tumor cells by inducing leukocyte antitumor cytotoxic activity. However, cytokines have been found to be significantly upregulated in many cancers, promoting cancer growth and progression within the tumor microenvironment (TME) [1,2]. Cytokines are classified into interleukins (IL), interferons (IFN), tumor necrosis factors (TNF), growth factors, hematopoietic factors, and chemokines. Accordingly, chemokines are proteins of relatively low molecular weight (8–14 kDa) and are classified into C-C motif (CC), C-X-C motif (CXC), C-motif (C), and CXC3 chemokines according to structural differences [3]. To date, around 50 types of chemokines have been identified [4,5]. The primary role of chemokines is to participate in leukocyte development, differentiation, and migration. However, chemokines are also involved in angiogenesis, wound healing, inflammatory diseases, and progression of malignancies, such as adult T-cell leukemia and many solid tumors [6,7,8,9,10]. Although chemokines exert antitumor effects, including induction of cytotoxic T cells into tumor tissues, they have also been known to assist cells that suppress tumor immunity, such as tumor-associated macrophages (TAM), myeloid-derived immune suppressor cells (MDSC), and regulatory T cells (Treg) [9,10,11]. Furthermore, chemokines can directly activate cancer cells via their corresponding receptors. However, the mechanisms of chemokine-related cancer activation have still remained mostly unclear. The present article highlighted the unique role of chemokine (C-C motif) ligand 20 (CCL20), which belongs to the CC chemokine family, and its specific receptor C-C chemokine receptor 6 (CCR6) in cancer progression.

### Basic Information Regarding CCL20 and CCR6

CCL20, which is known by several names, including macrophage inflammatory protein (MIP)-3α, Exodus-1, and liver and activation-regulated chemokine (LARC) [12,13], is expressed in various human tissues and immune cells and has been observed mainly in lymph nodes, lungs, and liver [12,14,15]. At the cellular level, endothelial cells, neutrophils, T helper (Th) 17 cells (Th17), B cells, natural killer cells, dendritic cells (DC), and macrophages have been reported to secrete CCL20 [16,17,18].

In 1997, Baba et al. reported that the specific receptor for CCL20 was CCR6 [19], a 7-transmembrane domain G protein-coupled receptor strongly expressed in the intestinal mucosa, lung mucosa, and lymph nodes, and highly in epithelial tumors [20,21,22,23,24]. At the cellular level, CCR6 is predominantly expressed on immune cells, such as Th17, Treg, CD8+ T cells, and B cells [25,26,27].

## 2. Role of the CCL20-CCR6 Axis in Cancer Progression and Tumor Microenvironment

Tumor growth can be regulated by not only internal tumor cell signals but also external factors. Studies have shown that tumor cells emit various signals to reconstruct a favorable environment for themselves. Generally, although normal immune cells attack and suppress cancer cells, some immune cells that infiltrate into cancer tissue lose their antitumor function and play a role in promoting tumor progression [28,29]. Therefore, a proper understanding of the TME and selection of therapeutic targets is important. However, the TME, which contains tumor cells, fibroblasts, immune cells (e.g., T cells), pericytes, endothelial cells, mesenchymal stem cells, adipocytes, red blood cells, and many other components, is considerably complex [30,31]. Furthermore, tumors develop in a different physiological environment. For instance, liver cancer develops in the presence of inflammation and fibrosis [32], which may not be that impactful in prostate and breast cancers. Although the cause of renal cell carcinoma (RCC) remains unknown in most cases, the RCC TME among patients undergoing dialysis and those with *Von Hippel–Lindau* (*VHL*) gene abnormality has been considered to differ significantly. Moreover, the TME can even differ among the same tumor types depending on the disease stage, thereby changing the expected target for effective treatment [33].

Signaling substances, such as cytokines released from tumor cells, stromal cells, and immune cells, are extremely important in the TME [34]. Accordingly, cytokines secreted by cancer cells affect a wide range of processes, such as autocrine signaling, paracrine signaling, and the circulatory system, and create a favorable environment for cancer cells by affecting various receptors. The main functions of chemokines include recruitment and signaling of immune cells, which cancer cells use to their advantage [35]. Although CCL20 exhibits antibacterial activity and may cause autoimmune diseases, such as rheumatoid arthritis, psoriasis, and inflammatory bowel disease, the evidence clearly suggests that the CCL20-CCR6 axis within the TME is strongly associated with cancer, with studies having reported several factors affected by the CCL20-CCR6 axis (Table 1).

### 2.1. Hepatocellular Carcinoma (HCC)

HCC, the main type of primary liver cancer, is the third leading cause of cancer-related deaths worldwide [56]. The main risk factors for HCC include chronic hepatitis B, hepatitis C virus infection, alcohol abuse, metabolic disorders, smoking, obesity, and diabetes. Partial hepatectomy has been the curative treatment option for patients with HCC who have an adequate liver function, although high recurrence rates have continued to be a concern [57,58]. The CCL20-CCR6 axis is also regarded as a key contributor to the progression of HCC [59]. Accordingly, Ding et al. found that tissue CCL20 expression was associated with tumor size, tumor number, vascular invasion, tumor differentiation, and tumor recurrence, and patients with high CCL20 expression had poorer recurrence-free survival and overall survival than those with low CCL20 expression [36]. Moreover, Yang et al. showed that bile duct tumor thrombosis and high CCL20 expression levels were poor prognostic factors for HCC [37]. Another study has revealed that CCL20 has been significantly upregulated in HCC tissues, while pretreatment serum CCL20 levels have been closely associated with patient survival [38]. The same study has found that CCL20 produced by HCC cells recruits CCR6+CD5+ B cells and induces angiogenesis to promote tumor growth and that blocking CCL20 suppressed angiogenesis. Overall, the highlighted studies suggest that the CCL20-CCR6 axis may be a novel target for HCC treatment.

### 2.2. Breast Cancer

Breast cancer, the most common cancer among women [60], can be classified into several subtypes according to the expression of estrogen receptor (ER), progesterone receptor (PR), human epidermal growth factor receptor 2 (HER2), and Ki-67. Among them, ER-, PR-, and HER2-breast cancers have been known as triple-negative breast cancer (TNBC) to have a poor prognosis. Recently, however, CCL20 has also been associated with poor breast cancer prognosis. Among patients with breast cancer, those with high CCL20 expression have significantly lower overall survival and metastasis-free survival. One study has shown that intraperitoneal administration of anti-CCL20 antibody has inhibited osteolytic breast cancer bone metastasis in a mouse model, and CCL20 has significantly enhanced cell invasion and secretion of matrix metalloproteinase (MMP) 2 and 9 in TNBC cell lines [40]. Chemotherapy resistance has been a major issue in the treatment of TNBC. CCL20 promotes self-renewal and maintenance of breast cancer stem cells and breast cancer stem-like cells by activating p65 nuclear factor kappa B (NF-κB) via protein kinase C or p38 mitogen-activated protein kinase. Furthermore, CCL20-stimulated NF-κB activation increases multidrug resistance 1 expression and promotes taxane extracellular excretion. The aforementioned results suggest that CCL20 is an important target for chemoresistance in breast cancer [41].

### 2.3. Colorectal Cancer (CRC)

Early diagnosis of CRC, which is common among both men and women [60], is important for improving prognosis, with CCL20 being a candidate biomarker. Accordingly, studies have shown that patients with CRC have significantly higher serum CCL20 and interkeukin-17 A (IL17A) levels than healthy individuals, and that receiver operating characteristic curve analysis combining CCL20 and IL17A is effective in distinguishing patients with CRC from healthy individuals [42]. Recurrence and metastasis in CRC have been the main causes of death among patients, while chemoresistance has remained a major therapeutic challenge. One study has shown that CCL20 expression level in CRC cells has been significantly increased among cases resistant to FOLFOX regimen chemotherapy and has been closely associated with worse survival [43]. Furthermore, CRC cells secrete CCL20 and recruit Treg into tumor tissues, enhancing their chemoresistance [43]. Based on available evidence, CCL20 can be considered a useful diagnostic marker and treatment target for CRC.

### 2.4. Pancreatic Cancer

Pancreatic cancer has one of the worst prognoses among carcinomas, with a 5-year survival rate of only 8%. Although several cancers have had extended survival rates due to advancements in cancer treatment, little improvement has been made for pancreatic cancer, given its rapid progression without symptoms and strong resistance to chemotherapy [60,61]. Considering that majority of patients cannot undergo curative resection, drug therapy is important. The transcriptional pathway NF-κB is particularly important, given its involvement in apoptosis resistance. One study has shown that CCL20 is the strongest target gene in TNF-related apoptosis-inducing ligand (TRAIL) resistance through the NF-κB subunit RelA [44]. Pancreatic cancer cells acquire TRAIL resistance through not only autocrine CCL20 but also paracrine recruitment of immune cells [44]. Among the immune cells, TAM expressing M2-type macrophage markers have been shown to promote tumor progression and suppress cytotoxic T-cell responses. Moreover, IL4-stimulated M2-type macrophages strongly express CCL20 and promote epithelial-mesenchymal transition (EMT) and pancreatic cancer cell invasion. Another study has shown that the CCL20-CCR6 axis has also promoted pancreatic cancer growth and liver metastasis in vivo in a mouse model [45].

### 2.5. Prostate Cancer

Inflammatory cytokines and chemokines released by macrophages in the prostate cancer microenvironment may signal via the androgen receptor to influence tumor progression [62]. It has been demonstrated that the interaction of infiltrating macrophages and prostate cancer cells mediates the hormone resistance of prostate cancer cells [63]. Expression levels of CCR6 in prostate cancer are associated with clinical and pathologic features of more advanced and aggressive prostate cancer status, such as local tumor volume, Gleason score, and lymph node metastasis [46]. One study has shown that the chemokine receptor CXCR4 stimulates the production of the chemokine CCL20 and that CCL20 stimulates the proliferation and adhesion to collagen of prostate cancer cells. Furthermore, overexpression of CCL20 in prostate cancer cells promotes growth and adhesion in vitro and increases tumor growth and invasiveness in the in vivo mouse model [47].

### 2.6. Lung Cancer

Tobacco kills nearly 6 million people each year, and 90% of the annual 1.59 million lung cancer deaths worldwide are caused by cigarette smoke. CCL20 is significantly upregulated by tobacco carcinogen nicotine-derived nitrosaminoketone [48]. Expression of lncRNA-u50535 is upregulated in lung cancer tissues and cell lines compared with normal tissues and cells, and Western blot and luciferase reporter gene assays have demonstrated that lncRNA-u50535 has also increased the translation and transcription of CCL20 in lung cancer cells [49]. Knockdown of lncRNA-u50535 decreases lung cancer cell proliferation and migration, induces G0/G1 phase arrest, and promotes cell apoptosis with decreased CCL20, CCR6, and phosphorylated ERK (pERK) levels [49]. CCL20/ERK signaling has been further investigated in lung adenocarcinoma samples obtained at surgery and cell lines and assessed for the expression, tissue localization, and production. CCL20 and CCR6 are found to be highly expressed in the majority of samples in the recurrence group (76 and 66%, respectively), and staining indexes of CCL20 and CCR6 in the recurrence group are 149.3 and 134.4, respectively, which are significantly higher than those in the non-recurrence group (57.2 and 58.0, respectively) [50]. In colony formation assay, ERK signaling and chemokine production have been measured to assess the responsiveness of the A549 cell line to CCL20 stimulation and have found the colony-forming phosphorylation [50]. Collectively, the findings suggest that CCR6 and CCL20 may serve a role in lung adenocarcinoma, leading to proliferation and migration via autocrine or paracrine mechanisms. The disruption of CCL20-CCR6 interactions may be a promising strategy for the treatment of cancer [50].

### 2.7. Cervical Cancer

Cervical cancer is the second most common cancer in women worldwide [64]. One study has shown that level of CCL20 in the cervical cancer tissues is significantly higher than that in non-tumor and normal control tissues and strongly positively associates with Th17 cells [51]. CCL20 is also predominantly expressed in the stroma of cervical cancer tissue and correlates with stromal infiltration of CD4+IL17+ cells and with advancing International Federation of Gynecology and Obstetrics (FIGO) stage [52]. Furthermore, cervical cancer cells instruct primary cervical fibroblasts to produce high levels of CCL20 and to attract CD4+IL17+CCR6+ cells [52].

### 2.8. Gastric Cancer

Upregulated CCR6 protein expression has also been observed in the gastric cancer tissues [24]. CT10 regulator of kinase like protein (CrkL) has been identified as a key regulator in EMT, and both CCR6 and CrkL are aberrantly expressed in gastric cancer specimens. The expression of CCR6 and CrkL is also significantly associated with metastasis, stage, and poor prognosis of gastric cancer. The knockdown of CrkL abrogates the CCL20-induced pERK, vimentin, N-cadherin, and MMP2 expression with decreased migration and invasion of gastric cancer cells [53].

### 2.9. Ovarian Cancer

In clinical ovarian cancer samples, high CCR6 expression on ovarian cancer cells positively correlates with cancer metastasis, leading to poor prognosis. Cisplatin-stimulated classically activated macrophages promote ovarian cancer cell migration by increasing CCL20 production, which activates its receptor CCR6 on ovarian cancer cells, triggering EMT. Pharmacological blockage of CCL20 on cisplatin-stimulated activated macrophages and siRNA-mediated inactivation of CCR6 on cancer cells effectively abrogate ovarian cancer cell migration induced by cisplatin-stimulated activated macrophages. These results suggest the CCL20-CCR6 axis as a potential therapeutic target to reduce chemotherapy-induced metastasis in advanced-stage ovarian cancer [54].

### 2.10. Renal Cell Carcinoma

RCC is the most common type of renal cancer, accounting for 3–4% of adult cancers in the United States [60]. Around 20–40% of patients with RCC develop recurrence and metastasis after initial surgery and require drug therapy [65]. Moreover, around 70% of all RCCs have been classified as clear cell RCC (ccRCC) derived from the proximal convoluted tubule [66]. Until the 1990s, immunotherapy, including IFN-α and IL2, had been the only drug therapy for ccRCC, which is immunogenic [67]. Thereafter, vascular endothelial growth factor receptor tyrosine kinase inhibitors had become the main treatment method. In recent years, however, immune checkpoint inhibitors had become the prominent treatment method for RCC, with immune tolerance again becoming the main concern in the treatment of RCC [68].

Several chemokine axes, such as the CCL2-CCR2 axis, the CCL3-CCR5 axis, and the CCL17/22-CCR4 axis, are reported to be involved in ccRCC [69,70,71,72,73]. The CCL20-CCR6 axis is also involved in the growth and progression of RCC. One study has revealed that among patients with RCC, those who have bone metastases have higher serum CCL20 levels than those who do not have bone metastases and that the CCL20-CCR6 axis perhaps be involved in RCC bone metastases [74]. Other studies have found that CCL20 secretion by RCC tissue induces invasion of Treg expressing high levels of CCR6 into RCC tissues, creating an optimal environment for itself [75,76]. TAM is an immune cell that greatly influences the TME along with Treg. A recent study has reported an interesting relationship between RCC and TAM via the CCL20-CCR6 axis. In vitro experiments have revealed that macrophage-like cells prepared from human monocytic leukemia cell line THP-1 promote RCC cell migration and that CCL20 substantially contributes to macrophage-induced RCC cell migration. Furthermore, AKT activation is involved in the promotion of RCC migration through the CCL20-CCR6 axis. Moreover, immunohistochemical staining of human RCC tissues has revealed that high CCR6 expression and macrophage infiltration are poor prognostic factors [55].

## 3. The Relationship between the CCL20-CCR6 Axis and Immune Cells

Immune cell infiltration into tumor tissues has been one of the most important effects of CCL20 on the TME. Specific immune cells recruited into tumor tissues undergo various changes when stimulated by cancer or stromal cells. Such changes favor the survival of cancer cells and promote worse prognosis for many patients. The following subsections have discussed the relationship between the CCL20-CCR6 axis and immune cells.

### 3.1. Tumor-Associated Macrophage

Macrophages, which are mainly responsible for innate immunity along with neutrophils, are the major components of the mononuclear phagocyte system, including myeloid progenitor cells and blood monocytes [77]. Tissue-resident and inflammatory macrophages are derived from bone marrow-derived monocyte progenitor cells circulating in the blood [78]. These progenitor cells extravasate toward target tissues, where they differentiate into mature macrophages and exert various functions depending on the environment. Macrophages can be generally classified into two subsets, M1-type and M2-type macrophages. M1-type macrophages are driven by IFN-γ, bacterial moieties, such as lipopolysaccharide, and toll-like receptor agonists and function as important cellular components involved in the inflammatory response and antitumor immunity [79]. Conversely, M2-type macrophages are activated by IL4 and IL10 and exert anti-inflammatory and sometimes tumor-promoting effects [79]. Growing evidence has shown that macrophages play a central role in the remodeling of both normal and diseased tissues through angiogenesis, basement membrane destruction, leukocyte infiltration, and immunosuppression. Among them, TAM present in the TME exhibits M2-type macrophage phenotype and is greatly involved in tumor progression. TAM has been shown to secrete angiogenic mediators, such as fibroblast growth factor and thymidine phosphorylase, which promote tumor angiogenesis [80]. They can also sense hypoxia due to vascular deprivation within the tumor and releases vascular endothelial growth factor A, a very potent pro-angiogenic factor, to enhance tumor survival and metastasis [80]. Furthermore, TAM produces MMPs and transforming growth factor-β that help with infiltration into tumor cells. Although TAM exerts many functions in cancer progression, reports have shown that chemokines, such as CCL20-CCR6 axis, are strongly involved.

Tumor immunity is deeply involved in the development of melanoma, considering that immune checkpoint inhibitors—nivolumab and ipilimumab—have been shown to be effective [81]. One study has shown that transplanting human melanoma cells into humanized mice and administering human CCL20 every 3 days double tumor growth [82]. High CCL20 expression in the tumor stroma is determined to be a poor prognostic factor, while CCL20 mRNA expression of TAM is higher than that of tumor cells, cancer-associated fibroblasts, T cells, and peripheral blood monocytes within tumor tissues [82]. CCR6-deficient mice have been found to have reduced incidences of CRC [83]. Moreover, CCL20 plays a role in the progression of CRC by inducing TAM into tumor tissues and promoting TNFα secretion in TAM [83]. Therefore, given that CCR6 deficiency downregulates tumor progression, the CCL20-CCR6 axis could be a therapeutic target [83]. The association between TAM and the CCL20-CCR6 axis has also been reported in pancreatic cancer. One study has shown that co-culturing with M2-type macrophages has enhanced the invasiveness of pancreatic cancer cell lines, whereas transfection with CCR6 RNA interference has significantly reduced the invasiveness of the cell lines [45]. The addition of recombinant CCL20 has enhanced EMT by increasing the expression of p-AKT, p-ERK, and N-cadherin and decreasing the expression of E-cadherin in pancreatic cancer cell lines [45]. TAM infiltration into RCC tissue has also induced EMT through CCR6-AKT pathway activation in RCC cells by secreting CCL20, thereby enhancing the progression of RCC [55].

### 3.2. Regulatory T Cells

Tregs are a subset of T cells that control the autoimmune response and express the endogenous forkhead box protein p3 (Foxp3)+, CD25+, CD4+ phenotype. Dysfunction and aberrant Treg expression have been closely associated with the development of autoimmune diseases. Mutations in the gene encoding the Treg-specific transcription factor Foxp3 inhibit Treg development and cause fatal diseases, such as immune dysregulation, polyendocrine deficiency, and X-linked syndrome [84]. On the contrary, a large number of Tregs infiltrate various cancer tissues and control many cells, such as T cells, B cells, natural killer cells, and DC, which often cause poor clinical prognosis. Furthermore, Tregs regulate various molecules, such as cytotoxic T-lymphocyte-associated antigen-4, IL2, and IL10, and promote tumor tolerance in the TME, making it a potential target for cancer treatment [84].

One study has shown that high CCR6 expression by circulatory Treg and directional migration toward CCL20 promote Treg migration into tumor tissue and that intratumoral CCL20 concentration and tumor-infiltrating Treg number are positively correlated [85]. Moreover, the number of tumor-infiltrating Treg is associated with cirrhosis and tumor differentiation, while the increase in tumor-infiltrating Treg is a poor prognostic factor among patients with HCC. Evidence has shown that Treg is induced by CCL20 and invades tumor tissue, but CCL20 may be derived from tumor cells, macrophages, and other stromal cells [86]. Marked infiltration by CCR6+ Treg has been observed in the CRC tissue, and it is possible that CRC cells and macrophages produce a large amount of CCL20. Injection of recombinant CCL20 into the tumor site has promoted Treg recruitment and tumor progression in CRC-transplanted mice [86]. In vitro experiments on RCC have revealed that renal cancer cell lines barely secrete CCL20, whereas macrophage-like cells markedly secrete the same [55]. Another study has shown that patients with RCC have increased Treg levels in the peripheral blood and tissues, with those having high Treg levels showing poor prognosis [87]. Treg is recruited by not only tumor cells but also TAM-derived CCL20, which may lead to cancer progression and poor prognosis among patients with HCC, CRC, and RCC.

### 3.3. T Helper 17 Cells

Th17 are Th lineage cells defined independently in 2005 from the subsets of Th1 and Th2 [88]. Th17 have been shown to secrete IL17, IL6, IL21, IL22, and TNF-α, which play important roles in autoimmune diseases and body defense responses. The primary physiological role of Th17 is to promote host defense against infectious agents and maintain barrier immunity on the skin and mucosal surfaces, such as intestines and lungs [89]. However, Th17 is also involved in inflammatory and autoimmune diseases and plays a detrimental role in psoriasis, rheumatoid arthritis, inflammatory bowel disease, asthma, and systemic lupus erythematosus [90].

Various chemokines, such as CCL2, CCL5, CCL17, CCL20, and CCL22, recruit Th17, depending on the situation [51,91,92]. Moreover, Th17 is often associated with tumors and has been reported in several cancer sites, including the kidney, prostate, colon, breast, hepatocytes, pancreas, and cervix [89]. In fact, reports have shown that Th17 infiltration into tumor tissue is a poor prognostic factor in certain cancers [93,94,95]. Moreover, one study has shown that CCL20 chemoattracts Th17 in vitro, while Th17, highly expressing CCR6, is significantly accumulated in cervical cancer tissue [51]. The expression level of CCL20 is significantly higher in tumor tissues than in normal control tissues, while CCL20 expression in tissues is positively correlated with Th17 number [51]. Another study has shown that IL6 derived from cervical cancer cells stimulates cervical fibroblasts and induces CCL20 secretion via the CCAAT/enhancer-binding protein β pathway [52]. CCL20 attracts CD4+IL17+CCR6+ Th17 and regulates the TME [52]. Therefore, the IL6-CCL20-CCR6 pathway could be a potential therapeutic target [52]. Conversely, Th17 has also been reported to improve the TME. In a large CRC cohort, tumor-infiltrating IL17-producing cells have not worsened clinical outcome and recruit highly cytotoxic CCR5+CCR6+CD8+ T cells via the release of CCL5 and CCL20 [96]. A study, which has evaluated Th1, Th2, Th17, and naive Treg ratios by flow cytometry from 131 patients with RCC and 36 healthy volunteers, has shown that patients with RCC have increased Th2 and Th17 and reduced Th1 cells and naive Treg in their peripheral blood [97]. In particular, as the tumor stage and grade progress, Th1 cells and naive Treg in the peripheral blood decrease, whereas Th2 and Th17 increase [97]. Furthermore, the number of tumor-infiltrating Foxp3+ Treg in tumor tissue increases with the progression of tumor stage [97]. This indicates that the balance between Th1 and Th2 cells is biased toward the Th2 profile, whereas the balance between Treg and Th17 is biased toward the Th17 profile in the peripheral blood of patients with RCC [97]. The host’s antitumor immunity could also be out of balance with Treg infiltration into the tumor tissue [97]. Thus, Th17 is undoubtedly important in tumor immunology, although its role is complex and bifactorial. As such, further research is needed.

### 3.4. Myeloid-Derived Suppressor Cells

Flow cytometric analysis of MDSC in the peripheral blood and tumor tissues of patients with RCC has shown a positive correlation with tumor grade and stage [98]. Furthermore, CCL2, IL17, and IL18, which are increased in peripheral blood and tumor tissues, may recruit MDSC into tumor tissues [98].

Gr1+CD11b+ cells have long been known to accumulate in the spleen and tumor tissue of mouse tumor models and exert strong immunosuppression [99]. However, these cells, collectively called MDSC in 2007, are heterogeneous and have various phenotypes [100]. MDSC can be broadly classified into granulocytic or polymorphonuclear (PMN-MDSC) and monocytic (M-MDSC). Morphologically, PMN-MDSC resembles neutrophils, while M-MDSC resembles monocytes. While other small subsets, such as immature MDSC (I-MDSC), do exist, most MDSCs are either PMN-MDSC or M-MDSC [101]. The main function of MDSC is immunosuppression, with T cells being their main target. MDSC inhibits tumor immunity through arginase 1, inducible nitric oxide synthase (iNOS), transforming growth factor-β, IL10, and cyclooxygenase 2 [101]. PMN-MDSCs are often defined as CD11b+CD14−CD15+ cells or CD11b+CD14−CD66b+ cells, while M-MDSCs are often defined as CD11b+CD14+HLA-DR-/loCD15-cells [101]. MDSCs are attracted to tumor tissues in response to various cytokines. Accordingly, CCL2 and CCL5 have been observed in M-MDSC, while CXCL1, CXCL5, CXCL6, CXCL8, and CXCL12 have been observed in PMN-MDSC [102]. MDSC suppresses CD8+ T cells, while tumor tissue-induced M-MDSC differentiates into TAM. Hence, tumor tissue is continuously recruited with TAM [103,104].

CCL2, which has been reported in breast cancer, gastric cancer, ovarian cancer, and CRC, is the most representative chemokine that induces MDSC in tumor tissue [105,106]. Tumor cell-produced CCL2 induces monocyte infiltration into tumor tissue and differentiates into TAM in the TME [107]. In severe combined immunodeficient (SCID) mice subcutaneously injected with vertebral-cancer of the prostate (VCaP) prostate cancer cell lines, systemic administration of anti-CCL2 neutralizing antibody has significantly slowed tumor growth and significantly suppressed angiogenesis and infiltration of CD68+ macrophages into tumor tissues [107]. Moreover, inhibition of the CCL2-CCR2 axis suppresses MDSC proliferation and migration, as well as TAM invasion, and contributes to TME improvement and patient prognosis. After evaluating MDSC and chemokines in tissues and peripheral blood of 48 RCC patients [108], Najjar et al. showed that the number of PMN-MDSC in tumor tissue was correlated with IL8 and CXCL5. Given that CXCR2 acts as the receptors for both IL8 and CXCL5, the effect of the CXCR2 blockade was examined, while combination therapy comprising anti-programmed cell death 1 (PD1) and CXCR2 blockade was performed on a Renca mouse model [108]. Accordingly, combination therapy caused a reduction in tumor weight and infiltration of CD4+ and CD8+ T cells into the tumor tissue [108]. Although no direct link between CCL20 and MDSC has been observed, MDSC is deeply involved in many tumors. Future studies may find an indirect role through other immune cells and cytokines.

## 4. Treatment for Cancers Targeting on the CCL20-CCR6 Axis

The CCL20-CCR6 axis promotes cancer progression, directly activating cancer cells and indirectly via inactivation of the tumor-immune system, and the blockade of the CCL20-CCR6 axis is thought as a promising target. However, clinically available drugs modulating the CCL20-CCR6 axis as anticancer agents are not available so far, and the development of such drugs is absolutely expected.

GSK3050002, a humanized IgG1κ antibody with high binding affinity to human CCL20, was administered in a first-in-human study to assess safety, tolerability, pharmacokinetics, immunogenicity, and biological activity. GSK3050002 was well tolerated, and no safety concerns were identified. The pharmacokinetics was linear over the dose range, with a half-life of approximately 2 weeks. The complex of GSK3050002/CCL20 increased in serum and blister fluid with increasing doses of GSK3050002. There were dose-dependent decreases in CCR6+ cell recruitment to skin blisters with maximal effects at doses of 5 mg/kg and higher, doses at which GSK3050002/CCL20 complex in serum and blister fluid also appeared to reach maximum levels [109].

Both CXCR3 and CCR6 chemokine receptors play crucial roles in the migration of pathological Th1 and Th17 cells during the course of certain inflammatory diseases. A fully humanized IgG-like bispecific antibody, which is simultaneously targeting both CXCR3 and CCR6, has been developed. This bispecific antibody binds to both chemokine receptors and effectively blocks chemotaxis of these cells and induces specific antibody-dependent cell-mediated cytotoxicity in vitro [110].

ASN002 is an oral inhibitor of the Janus kinase/spleen tyrosine kinase signaling pathways, targeting several cytokine axes, including CCL20. ASN002 has been studied as a phase 1B clinical trial in 36 patients with atopic dermatitis. ASN002 has shown strong inhibition of IL-17-mediated CCL20 release in keratinocytes [111].

These drugs targeting the CCL20-CCR6 axis display a potent interventional approach for the treatment of inflammatory and autoimmune diseases; however, the application of these promising drugs for cancer patients may need more basic investigations and clinical trials.

## 5. Conclusions and Future Outlook

**Figure 1:**
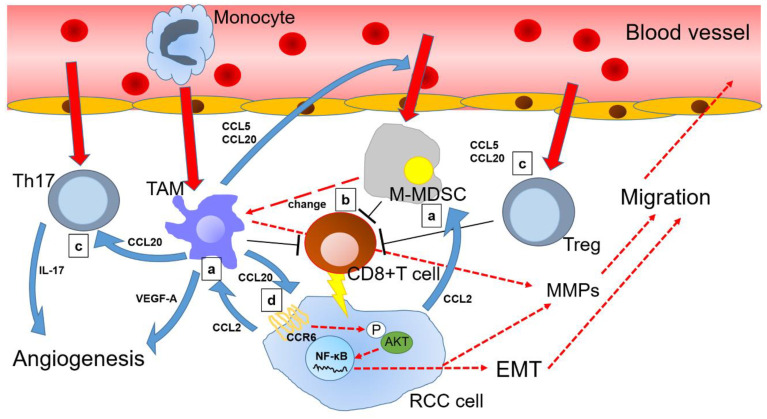
Overview of chemokine (C-C motif) ligand 20 (CCL20) and other related chemokine signals in the renal cell carcinoma microenvironment: (**a**) Renal cell carcinoma (RCC) cells secrete CCL2 to guide tumor-associated macrophage (TAM) and monocytic myeloid-derived suppressor cells (M-MDSC) toward tumor microenvironment (TME); (**b**) M-MDSC suppress CD8+ T cells together with TAM, while a portion of M-MDSC changes into TAM, which continues to reside in the TME; (**c**) TAM mobilizes T helper 17 cells (Th17) and regulatory T cells (Treg) via the CCL20-chemokine receptor 6 (CCR6) axis to enhance angiogenesis and suppress antitumor immunity, thereby indirectly promoting RCC progression; (**d**) Furthermore, CCL20 binds to CCR6 of RCC cells and induces epithelial-mesenchymal transition (EMT) via AKT activation of RCC cells, thereby directly promoting cancer progression. Solid red arrows indicate cell movement. Dotted red arrows indicate changes in cell status. Blue arrows indicate protein secretion. Long T shaped bars indicate functional inhibition.

Despite CCR6 being the only CCL20 receptor, its role in TME is considerably complex. This is because the cytokine network is intricately involved in and controls tumor progression. Interestingly, CCL20 and CXCL8 have been shown to jointly, but not separately, induce EMT in CRC cells. Moreover, co-expression of CCL20 and CXCL8 is negatively correlated with E-cadherin expression in CRC tissues, confirming two synergistic roles for EMT in CRC cells [112]. CCL2 from RCC tissues may induce TAM [71], secreting CCL20 and inducing Treg and Th17 [55,113]. Th17, in turn, invades RCC tissues and enhances IL8 expression in tumor tissue by releasing IL17 [114]. The IL8-CXCR1 axis activation has been associated with poor prognosis among patients with ccRCC [115]. Thus, cytokines, including chemokines and immune cells, are involved in various networks across many cancers, including RCC (Figure 1). More in vivo experiments and clinical trials are certainly needed to better determine the critical roles of the CCL20-CCR6 axis in cancer development.

## Figures and Tables

**Table 1:** Tumor promoting effects of CCL20 within the tumor microenvironment.

Cancer Types	Specimens	Factors	Description Contents	References
HCC	Tissue	-	High expression of CCL20 in tumor tissues exacerbates recurrence rate and survival among patients with HCC.	[36]
HCC	Tissue	BDTT	CCL20 is highly expressed in BDTT and is a poor factor in HCC prognosis.	[37]
HCC	Blood Tissue Cell lines Mouse model	B cells	Tumor cell-derived CCL20 interacts with CCR6-highly expressed CD19+CD5+ B cells to promote HCC progression through enhanced angiogenesis.	[38]
HCC	Blood Tissue	STAT3	Tumor cells transfected with STAT3 siRNA show significantly lower CCL20 expression than control tumor cells.	[39]
Breast cancer	Tissue Cell lines Mouse model	HuR	HuR enhances the invasion of cancer cells through CCL20 and GM-CSF.	[40]
Breast cancer	Blood Tissue Cell lines Mouse model	NF-κB	CCL20 expression in TNBC induces taxane resistance via the NF-κB pathway.	[41]
CRC	Blood Tissue	IL17A	Serum CCL20 and IL17A levels are identified as independent prognostic markers for CRC.	[42]
CRC	Blood Tissue Cell lines Mouse model	Treg NF-κB	CRC cell-secreted CCL20 can recruit Treg to promote chemoresistance via FOXO1/CEBPB/NF-κB signaling.	[43]
Pancreatic cancer	Blood Cell lines	NF-κB	Pancreatic cancer cells also acquire TRAIL resistance by recruiting immune cells with CCL20.	[44]
Pancreatic cancer	Blood Cell lines Mouse model	TAM AKT ERK	M2 macrophages secrete CCL20 and increase pancreatic cancer cell invasiveness.	[45]
Prostate cancer	Tissue	-	Strong CCR6 expression in prostate cancer tissue is a poor prognostic factor.	[46]
Prostate cancer	Tissue Cell lines Mouse model	CXCL12 CXCR4	CXCL12 derived from cancer-associated fibroblasts acts on CXCR4 of prostate cancer cells, and the prostate cancer cells overexpress CCL20 and progress.	[47]
Lung cancer	Tissue Cell lines Mouse model	Nitrosaminoketone	Nitrosaminoketone induces the production of CCL20 and promotes the proliferation and migration of lung cancer cells. High expression of CCL20 in lung cancer tissues is a poor prognostic factor.	[48]
Lung cancer	Tissue Cell lines Mouse model	lncRNA-u50535 ERK	lncRNA-u5053 upregulates CCL20 expression in lung cancer cells, and lung cancer cells enhance proliferation and migration via the CCL20/CCR6/ERK axis.	[49]
Lung cancer	Tissue Cell lines	ERK	CCL20 and CCR6 are highly expressed in recurrent lung cancer tissue. Lung cancer cells activate the CCL20/CCR6/ERK axis via autocrine or paracrine mechanisms to promote proliferation and migration.	[50]
Cervical cancer	Tissue Cell lines	Th17	Th17 cells are recruited into tumor tissues preferentially through CCL20-CCR6 pathway.	[51]
Cervical cancer	Tissue Cell lines	Th17	IL6 derived from cervical cancer cells stimulates cervical fibroblasts and induces CCL20 secretion.	[52]
Gastric cancer	Tissue	-	The expression of CCR6 in gastric cancer cells is upregulated as compared with that in normal tissues, and the high expression of CCR6 is an independent poor prognostic factor.	[24]
Gastric cancer	Tissue Cell lines	CrkL ERK	Gastric cancer cells are stimulated by the CCL20/CCR6-CrkL-ERK1/2 axis to enhance invasion, and the high expression of CCR6 and CrkL in cancer tissues is an independent poor prognostic factor.	[53]
Ovarian cancer	Tissue Blood Cell lines	Macrophage Cisplatin	Macrophages stimulated by cisplatin produce CCL20 and enhance the migration of ovarian cancer cells.	[54]
RCC	Tissue Cell lines	TAM AKT	TAM enhances RCC cell migration through the CCL20-CCR6 pathway.	[55]

HCC, hepatocellular carcinoma; BDTT, bile duct tumor thrombus; HuR, human antigen R; GM-CSF, granulocyte-macrophage colony-stimulating factor; TNBC, triple-negative breast cancer; STAT3, signal transduction and activator of transcription 3; CRC, colorectal cancer; Treg, regulatory T cells; FOXO1, forkhead box protein O1; CEBPB, CCAAT enhancer-binding protein beta; NF-κB, nuclear factor kappa B; IL17, interleukin 17; ERK, extracellular signal-regulated kinase; CXCL12, chemokine (C-X-C motif) ligand 12; CXCR, C-X-C chemokine receptor type 4 TRAIL, human tumor necrosis factor (TNF)-related apoptosis-inducing ligand; TAM, tumor-associated macrophages; Th17, T helper 17 cells; CrkL, CT10 regulator of kinase like protein.

## Abbreviations

CC	C-C motif
CCL	Chemokine (C-C Motif) Ligand
CCR	C-C Chemokine Receptor
CrkL	CT10 Regulator of Kinase Like Protein
CRC	Colorectal Cancer
CXC	C-X-C Motif
CXCL	Chemokine (C-X-C Motif) Ligand
CXCR	C-X-C Chemokine Receptor
DC	Dendritic Cells
EMT	Epithelial-Mesenchymal Transition
ER	Estrogen Receptor
ERK	Extracellular Signal-Regulated Kinase
Foxp3	Forkhead Box Protein P3
HER2	Human Epidermal Growth Factor Receptor 2
IFN	Interferon
IL	Interleukin
IL17A	Interkeukin-17 A
iNOS	Inducible Nitric Oxide Synthase
LARC	Liver and Activation-Regulated Chemokine
MDSC	Myeloid-Derived Immune Suppressor Cells
MMP	Matrix Metalloproteinase
NF-κB	Nuclear Factor-Kappa B
PD1	Programmed Cell Death 1
PR	Progesterone Receptor
RCC	Renal Cell Carcinoma
SCID	Severe Combined Immunodeficient
TAM	Tumor-Associated Macrophages
Th	T Helper Cells
TME	Tumor Microenvironment
TNBC	Triple-Negative Breast Cancer
TNF	Tumor Necrosis Factors
TRAIL	TNF-Related Apoptosis-Inducing Ligand
Treg	Regulatory T Cells
VCaP	Vertebral-Cancer of the Prostate
